# Mild cognitive impairment (MCI) and dementia in a sample of adults in the city of Bogotá

**DOI:** 10.1590/1980-57642016dn11-030008

**Published:** 2017

**Authors:** Olga Lucia Pedraza, Ana Maria Salazar Montes, Fabio Alexander Sierra, Maria Camila Montalvo, Yolanda Muñoz, Jose Miguel Díaz, Angela Lozano, Cesar Piñeros

**Affiliations:** 1 Interdisciplinary Group on Memory, Hospital Infantil Universitario de San José (HIUSJ).; 2 Department of Neurosciences, School of Medicine, Fundación Universitaria de Ciencias de la Salud (FUCS) Bogotá, Colombia.; 3 Department of Psychology, El Bosque University.; 4 Department of Epidemiology and Research (FUCS).; 5 Department of Nursing (FUCS).

**Keywords:** dementia, mild cognitive impairment, prevalence, demência, comprometimento cognitivo leve, prevalência

## Abstract

The low prevalence of dementia described in communities is likely due to the low sensitivity of screening tests and an absence of evaluation by specialists.

**OBJECTIVE:**

To estimate the prevalence of mild cognitive impairment (MCI) and dementia in adults older than 50 years.

**METHODS:**

A two-phase, cross-sectional study was conducted by specialists to evaluate cognition and associated demographic risk factors in 1,235 independent community-dwelling adults from Bogotá. In Phase I, screening was performed using the MMSE and MoCA tests. In Phase II, after application of a comprehensive neuropsychological battery with neurologic and psychiatric evaluations, a cognitive diagnosis was established by consensus.

**RESULTS:**

The prevalence found for MCI was 34% and for dementia was 23%. MCI was associated with incomplete high school, OR=1.74 (95%CI=1.23-2.45), and with an age of 70-79 years, OR=1.93 (95%CI=1.47-2.53). A total of 73% of MCI cases were amnestic. Dementia was associated with incomplete primary education, OR=8.98 (95%CI=5.56-14.54), complete primary education, OR=6.23 (95%CI=3.70-10.47), and age older than eighty years, OR=3.49 (95%CI=2.23-5.44).

**CONCLUSION:**

The prevalence of dementia found was greater than the rates reported in previous studies. Low educational level was the main risk factor for cognitive impairment and should be considered in strategic planning for the local health system.

## RESUMO

A baixa prevalência de demência relatada em comunidades deve ser devida ao emprego de testes de rastreio de baixa sensibilidade e à falta da avaliação por especialistas.

**OBJETIVO:**

Estimar a prevalência de comprometimento cognitivo leve (CCL) e demência em adultos com idade superior a 50 anos.

**MÉTODOS:**

Um estudo transversal de duas fases realizado por especialistas, avaliando a cognição e os fatores de risco demográficos associados, com 1.235 adultos autônomos da comunidade em Bogotá. Em uma Fase I, foram realizados os testes de rastreio MEEM e MoCA. Na Fase II, após uma ampla bateria neuropsicológica com avaliações neurológicas e psiquiátricas, foi estabelecido um diagnóstico cognitivo por consenso.

**RESULTADOS:**

A prevalência encontrada de CCL foi de 34% e de demência, de 23%. CCL foi associado a ensino médio incompleto, OR=1,74 (IC 95%=1,23-2,45) e idade entre 70-79 anos, OR=1,93 (IC 95%=1,47-2,53). Entre os casos de CCL, 73% eram amnésticos. A demência foi associada a ensino fundamental incompleto, OR=8,98 (IC 95%=5,56-14,54), ensino fundamental completo, OR=6,23 (IC 95%=3,70-10,47) e idade superior a oitenta anos, OR=3,49 (IC 95%=2,23-5,44).

**CONCLUSÃO:**

A prevalência de demência encontrada é maior do que a relatada em estudos prévios. O baixo nível educacional foi o principal fator de risco para declínio cognitivo e deve ser considerado no planejamento estratégico do nosso sistema de saúde.

## INTRODUCTION

World aging is a major concern, and Latin America, including Colombia, is aging faster than the Old World and the U.S.A.^1^ According to data from the World Health Organization (WHO) published in 2012, by 2050, four out of five people aged 60 or older will reside in developing countries.^2^ Under these circumstances, the occurrence of chronic and disabling diseases must be anticipated by health care systems. In 2013, Alzheimer's Disease International (ADI) predicted an increase in the number of elderly dependents, set to triple from 101 million in 2010 to 277 million by 2050. Based on this scenario, almost half will suffer from dementia associated with Alzheimer's disease or another type of dementia, and 71% of patients with dementia will reside in middle or low-income countries.^3^^,^ 4 According to reports published in 2010 by the Latin American and Caribbean Demographic Center (CELADE), the Economic Commission for Latin America and the Caribbean (ECLAC-CEPAL), and the Ministry of Health of Colombia, the projected aging index of the population in Colombia will increase from 20.9% in 2000 to 116.1% by 2050.^5^

The prevalence of dementia worldwide has been reported to be approximately 5 to 6% for most regions of the world and 8.5% in Latin America.^4^ The dementia prevalence in previous Colombian studies ranges from 6 to 23%. The EPINEURO study published by Pradilla et al. in 2003, reported one of the lowest rates of dementia worldwide, at 13.1‰ of the general population.^6^ In the study, the Mini-Mental State Examination (MMSE) was used as a screening instrument for dementia, which has low sensitivity for this task.^7^^,^ 8 In a second study published in 2013, Diaz et al. reported a 6% prevalence of cognitive impairment in 317 elderly people from the city of Manizales (Caldas) using a combination of several different, non-validated screening instruments.^9^ Another study published in 2006 by Gooding et al. found a 23% prevalence of dementia in the elderly population of Huila, in a two-phase study using different instruments and a broader protocol.^10^

Mild cognitive impairment (MCI) is considered an intermediate state between the cognitive changes associated with normal aging and mild dementia.^11^ Its prevalence has been reported to be between 10 and 20% in people older than 65 years.^12^ The annual rate of progression to MCI in normal subjects has been estimated at between 1 and 4% annually, and subjects with MCI have an annual risk of 12% of developing dementia.^4^ In 2010, Henao et al. published a study that evaluated 848 subjects older than 50 years in Medellin (Colombia) and reported a 9.7% prevalence of amnestic MCI with predominance in men; in the study, the CERAD instrument, including the MMSE, was used in addition to other more sensitive instruments validated by the Antioquia Neurosciences Group.^13^^,^ 14

There are many controversial studies that have analyzed the risk factors associated with MCI and dementia, such as increased age, gender, educational level, family history, and the APOE genotype.^15^^-^ 18 Also, cardiovascular risk factors, especially in the middle-aged population, can decrease and change during the course of dementia if controlled.^19^^-^ 24 Our study aims to determine the prevalence of, and the demographic risk factors associated with, MCI and dementia in a sample of the adult population older than 50 years living in different locations of Bogotá.

## METHODS

### Design.

A cross-sectional study was conducted in two phases. The data was collected between 2012 and 2014.

### Participants.

A minimum sample size of 1,095 adults over the age of 60 distributed across the different districts in the city was calculated. In addition to this sample, a group of adults aged 50-59 years who expressed willingness to participate in the study was also included.

Non-probability sampling was conducted involving all of the participants who agreed to be included in the study after receiving an invitation by letter sent to parish centers, retiree groups, cultural centers, and local and community action committees located in different districts of Bogotá. The inclusion criteria included independent and autonomous subjects over the age of fifty. The following exclusion criteria were used: prior history of psychiatric illness, subjects displaced due to violence, and subjects belonging to indigenous, illiterate, or institutionalized populations.

### Procedure and instruments.

**Figure 1:**
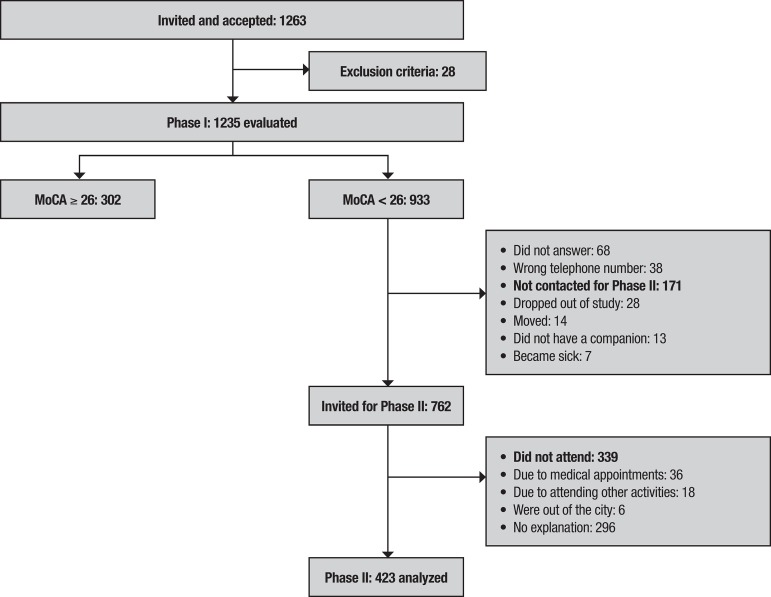
Flowchart of inclusion of subjects in the study

After an informed consent form was signed by the participants, the procedure was conducted in 2 phases as follows:

*Phase I.* 1,235 subjects were included (Figure 1) and groups no larger than 20 elderly adults, accompanied by a relative or caregiver, were invited to participate in a 1-hour evaluation at community centers in the neighborhoods of the respective location. Each research subject and their companion were seated facing the trained evaluator. A medicine or nursing evaluator administered questionnaires on: sociodemographic aspects, clinical history and risk factors, clinical criteria of the DSM-IV for dementia, and memory complaint^25^^,^ 26 and functionality (Barthel index) questionnaires.^27^ The caregiver answered two additional questionnaires related to the participant, namely, the subjective memory complaints questionnaire (SMCQ) and the Zarit Burden Interview.^26^^,^ 28 Additionally, a psychologist administered the subjective memory complaints questionnaire (SMCQ),^26^ MoCA Test,^29^ MMSE,^7^ and the Yesavage Depression Scale to the participants.^30^

To conclude Phase I, the different scores of the tests were obtained and calculated. Subjects scoring <26 on the MoCA and <24 on the MMSE, were invited to take part in the Phase II evaluation. In Phase II, the differences in results on these tests were clarified through assessment by consensus of specialists . During the Phase I evaluation, weight and size of participants were measured to calculate body mass index (BMI), and a peripheral blood sample was collected for DNA extraction and *APOE* genotyping.^31^

*Phase II.* For this evaluation, the participant was invited by telephone, within 2-6 months of Phase I, to participate in Phase II together with their relative or caregiver. The evaluation of 423 subjects (Figure 1) was conducted at the hospital by researchers, specialists in neurology, psychiatry or neuropsychology or medical residents in family medicine trained to perform the Phase II protocol. The memory clinic protocol was applied to the subject comprising a review of the medical history, a neurological and psychiatric exam, and blood pressure measurements. Additionally, the Hachinski Ischemic Scale and the Clock-Drawing Test were administered by the medical group.^32^^,^ 33 The neuropsychologic battery included: the Grober-Buschke memory test,^34^ the language fluency test,^35^ the 64-item naming test,^36^ the visuoconstructional and executive function tests, including the Rey-Osterrieth Complex Figure Drawing Test,^37^ and the TMT-A.^38^ The psychiatric Battery also included: the Kertesz Frontal Behavioral Inventory,^39^ and the Lawton (IADL) Scale,^40^ administered to the relative or caregiver, collecting information about the participant. Finally, the cognitive state of each participant was determined by consensus of the medical and psychologists group, and was classified as normal cognition, MCI, or dementia, according to the different clinical results. The diagnosis of dementia was established according to the DSM-IV criteria^25^ and of MCI according to the Petersen criteria.^11^ The MMSE and MoCA tests were again administered to each participant in a manner that was independent and blinded to the diagnosis; the cut-off point proposed for the MoCA test was used.^41^

### Statistical analysis.

To calculate the minimum sample size of individuals older than sixty needed in this study to provide a valid estimation of the prevalence of dementia, the number of elderly in Bogotá, and the percentages of dementia prevalence described in the literature by age categories, were considered. According to the 2005 census, elderly represented 6.7% of the total population. A type I error was assumed for 0.05 and a power of 80%.

To describe the sociodemographic and clinical characteristics of the participants, absolute and relative frequencies were used in the case of qualitative variables, and measures of central tendency with dispersion were used in the case of quantitative variables.

Prevalence of the MCI and dementia sub-types was described by absolute and relative frequencies. Sensitivity and specificity were calculated by age and by educational level per diagnosis established by consensus and MoCA test scores.

Characterization of the associated risk factors for MCI and dementia was conducted by means of logistic regression; the dependent variable was the diagnosis (MCI versus normal or dementia versus normal), and the independent variables were the factors described in the literature (comorbidities, age, gender and educational level); the OR was calculated with a confidence interval of 95%. Analyses were performed using the Stata 12^®^ statistical package.

This study was approved by the Ethics and Research Committees of the School of Medicine of the Health Sciences Foundation (FUCS) and met the requirements of the Declaration of Helsinki and resolution 8430 of 1993 on ethics in research on human beings in Colombia.

## RESULTS

In Phase I, 1,235 adults from 19 districts in Bogota were evaluated. A flowchart outlining the process of recruitment and evaluation of the patients in the two phases is shown in Figure 1. Seventy-five percent (75%) of the participants were women; the mean age of the group was 68 years (SD 8.6), and the mean educational level was 8 years (SD 6.0). A total of 325 (26%) subjects had an MMSE score <24, and 933 (76%) subjects had an MoCA test score <26. The latter group included all subjects with an MMSE score <24. There were 302 subjects (24%) with an MoCA test score ≥26, and these were considered as having normal cognition (Table 1). Of the 933 subjects with an MoCA score <26, only 762 could be contacted and agreed to participate in Phase II (Figure 1).

**Table 1:** Sociodemographic data of subjects with normal MoCA (≥26) in Phase I, and of subjects attending Phase II (MoCA<26), with cognitive diagnosis established by consensus.

Sociodemographic variable		MoCA ≥ 26 Phase I		MoCA <26 Phase II			
		Normal (n=302)		Normal (n=152)	MCI (n=166)	Dementia (n=105)	Total (n=423)
Gender	Female	218 (72.2)		113 (74.3)	120 (72.3)	82 (78.1)	315 (74.4)
	Male	84 (27.8)		39 (25.7)	46 (27.7)	23 (21.9)	108 (25.5)
Age	50 to 59	79 (26.2)		29 (19.1)	13 (7.8)	1 (1.0)	43 (10.1)
	60 to 69	157 (52.0)		77 (50.7)	70 (42.2)	28 (26.7)	175 (41.3)
	70 to 79	56 (18.5)		37 (24.3)	66 (39.8)	48 (45.7)	151 (35.6)
	80 and older	10 (3.3)		9 (5.9)	17 (10.2)	28 (26.7)	54 (12.7)
Educational level	Primary	48 (15.9)		73 (48.0)	96 (57.8)	91 (86.7)	260 (61.4)
	Incomplete high school	50 (16.6)		20 (13.2)	33 (19.9)	11 (10.5)	64 (15.1)
	Complete high school	40 (13.2)		15 (9.9)	14 (8.4)	2 (1.9)	31 (7.3)
	Higher education	164 (54.3)		44 (28.9)	23 (13.9)	0 (0.0)	67 (15.8)

MCI: Mild Cognitive Impairment. Data are presented as n (%).

Ultimately, in Phase II only 423 subjects were evaluated; 315 (74%) were women. The consensus diagnosis of the cognitive state of this group, revealed that 152 (36%) subjects were cognitively normal, and 166 (39%) subjects presented MCI according to the Petersen criteria, had a memory complaint corroborated by the companion, and neuropsychological test scores 1.5 SD below expected, with preserved functionality and no dementia. A further 105 (25%) subjects presented dementia (DSM-IV criteria) (Table 1).

Of the 510 subjects not participating in Phase II, 171 could not be contacted, and 339 did not attend the scheduled appointment (Figure 1); to establish a diagnosis in this group, their Phase I results were analyzed regarding age, education, criteria for MCI and dementia, medical history and sociodemographic aspects obtained from the questionnaires, and the scores obtained for each item on the MMSE and MoCA tests using the cut-off point from the MoCA validation (MCI <22 and dementia <18),^41^ and on the SMCQ(s), Yesavage scale, Barthel Index and responses given by the companion for the SMCQ(c). In an analysis, the means on the MoCA and MMSE were also compared for subjects attending and not attending Phase II, revealing no difference.

For the total group of 1,235 subjects, normal cognitive function was found in 43% of the adults, 34% were diagnosed with MCI and 23% with dementia (Table 2). These findings with regards to MCI and dementia are higher than rates reported by previous authors.^4^^,^ 6 ^,^ 9 ^,^ 15

**Table 2:** Prevalence of MCI and dementia by gender, age and educational level in the 1,235 subjects.

Sociodemographic variable		Diagnosis*			Total (n=1235)
		Normal n=534 (43%)	MCI n=421 (34%)	Dementia n=280 (23%)	
Gender	Female	389 (72.8)	315 (74.8)	225 (80.4)	929 (75.2)
	Male	145 (27.2)	106 (25.2)	55 (19.6)	306 (24.8)
Age	50 to 59	124 (23.2)	49 (11.6)	12 (4.3)	185 (15.0)
	60 to 69	269 (50.4)	161 (38.2)	89 (31.8)	519 (42.0)
	70 to 79	116 (21.7)	173 (41.1)	111 (39.6)	400 (32.4)
	80 and older	25 (4.7)	38 (9.0)	68 (24.3)	131 (10.6)
Educational level	Primary	148 (27.7)	215 (51.1)	221 (78.9)	584 (47.3)
	Incomplete high school	78 (14.6)	84 (20.0)	34 (12.1)	196 (15.9)
	Complete high school	68 (12.7)	48 (11.4)	10 (3.6)	126 (10.2)
	Higher education	240 (44.9)	74 (17.6)	15 (5.4)	329 (26.6)

MCI: Mild Cognitive Impairment. Data are presented as n (%).

* Each study participant was classified as a subject having normal cognition, MCI, or dementia, according to the different clinical results, the evaluation and consensus diagnosis or values on the MoCA test according to cut-off point in subjects not-attending Phase II. The diagnosis of dementia was established according to the DSM-IV criteria and Petersen criteria for MCI.

Regarding MCI, the frequency of amnestic MCI was 73%, proving the most frequent type for both genders. Non-amnestic MCI tended to occur in younger individuals (50 to 69 years), and in subjects with low education (Table 3).

**Table 3:** Prevalence of amnestic and non-amnestic MCI by gender, age and educational level.

Sociodemographic variable		Amnestic n (%)	Non-Amnestic n (%)	Total N (100%)
Total		309 (73.4)	112 (26.6)	421
Gender	Female	219 (69.5)	96 (30.5)	315
	Male	90 (84.9)	16 (15.0)	106
Age	50 to 59	34 (69.4)	15 (30.6)	49
	60 to 69	117 (72.7)	44 (27.3)	161
	70 to 79	131 (75.7)	42 (24.3)	173
	80 and older	27 (71.0)	11 (29.0)	38
Educational level	0 to 4 years	96 (72.7)	36 (27.3)	132
	5 years	56 (67.5)	27 (32.5)	83
	6 to 10 years	61 (73.5)	22 (26.5)	83
	11 years	37 (75.5)	12 (24.5)	49
	12 to 14 years	17 (77.3)	5 (22.7)	22
	15 to 16 years	7 (70.0)	3 (30.0)	10
	More than 17 years	35 (83.3)	7 (16.7)	42

Regarding the analysis of risk factors, dementia was associated with a low educational level, such as incomplete primary education, OR=8.98 (95% CI=5.56-14.54), complete primary education, OR=6.23 (95% CI=3.70-10.47), incomplete high school, OR=2.50 (95% CI=1.35-4.59), and with age older than 80 years, OR=3.49 (95% CI= 2.23-5.44).

## DISCUSSION

Bogotá is a metropolis that has always had a large Colombian population, which migrated or was displaced because of socioeconomic or political circumstances. According to the secretary of planning of Bogotá, the population in 2016 was 7,878,783 persons. Older adults represented 11.4% of the population and had an average of 5.5 years of education. A prevalence of dementia of 23% was found, greatly surpassing the 13.1% and 6% reported for Colombia.^6^^,^ 9 Recently, Brazil reported a higher prevalence of dementia of 17.5%,^42^ compared to previous studies, in the elderly population (average 71 years) together with low levels of education (mean 4.9 years).^41^ Peru reported a dementia prevalence of 6.85%,^43^ while the figure estimated for Latin America is 8.5%.^4^ These studies used a wide variety of instruments, some of which were validated by the research groups themselves while others were validated by studies conducted in countries with similar languages. Unfortunately, in previous studies, the MMSE has been used as a screening test for dementia in spite of its low sensitivity.^6^^,^ 8

Furthermore, the marked differences published in these studies on the prevalence of dementia may be attributed to the different diagnostic criteria, study populations, methodologies and instruments used. This holds true particularly for the screening tools used to select the subjects suspected of having cognitive impairment, such as the MMSE, which are most likely responsible for the low prevalence described in many of these studies. Erkinjuntti et al. reported a prevalence of dementia of 3.1% when applying the ICD-10 criteria and of 29.1% when using the DSM-III criteria.^44^ In his 10/66 study, Prince proposed the use of instruments that have been validated in developing countries, as well as clinical criteria such as the DSM-IV, the CIE-10, but also the MMSE from the CERAD battery, widely used in screening studies for the detection of dementia, but also having low sensitivity.^45^ The prevalence of dementia using the standardized DSM-IV and the instruments employed by the 10/66 group (CERAD) was 6.4% in Cuba and 11.7% in the Dominican Republic.^46^^,^ 47 The 10/66 group study administered the Community Screening Interview for Dementia (CSID), CERAD and Geriatric Mental State (GMS);^45^ but, a study comparing results on the prevalence of dementia for the 10/66 instruments versus the DSM-IV revealed some major differences in the results for each instrument.^48^ We believe that our use of the MoCA test, an instrument with high sensitivity and specificity, adopting the cut-off point determined by the author (<26), in a community population with a low educational level, led to a significant number of false positives (18% in the present sample), subsequently selected for Phase II. However, the application of the memory clinic protocol, allowed a correct diagnosis classification with the consensus diagnosis, thereby reducing the bias of the MoCA test in low educated individuals, as was the case with the 423 subjects evaluated in Phase II. In an interim analysis, we compared the means of MoCA between subjects attending and not- attending Phase II, and found no significant differences. The 510 subjects who did not participate in Phase II were evaluated using our validated cut-off point for the MoCA test, analyzing the memory complaint questionnaires and the clinical criteria from their history.^41^

Our results confirm the findings described by Gooding et al., 2006, who conducted a study screening with an extended version of the MMSE and an extended neuropsychological battery applied by specialists in a Neiva population with a low educational level, reporting a dementia prevalence of 23.6%.^10^

The MCI prevalence of 34% in our sample also greatly exceeded the 9.4% prevalence reported by Henao et al.. This difference may be due to a bias in the sample of the Henao study, which would not be represented in the community population from Medellín.^13^ However, the prevalence of MCI has been reported to be as great as 42% in other community studies.^14^

The MCI found in our study showed that the amnestic type was the most common form for both genders and that the non-amnestic form was not more frequent in men.^12^ A longitudinal follow-up of the population with normal cognition or with MCI will allow not only a prevention intervention, but an understanding of the relationship of cognitive impairment with the risk factors, different to the level of education.

Finally, late-onset dementia has been associated with carriers of genetic factors, such as being homozygous or heterozygous for the *APOE4* allele. This allele is considered to be responsible for 50% of late-onset Alzheimer dementia (AD).^49^ In Colombia, an association between *APOE4* and late-onset AD with a risk of 5.1% has been reported;^50^ currently, we are evaluating the association of *APOE4* with cognitive impairment among the subjects from the present study sample, and the association of their cardiometabolic risk factors are discussed in a recent paper.^51^

Given the impact of the high prevalence of cognitive impairment in the adult population of Bogotá, we must take into account our local data when establishing priorities and economic resources for health prevention policies; this could also be done by the research groups of universities.

To conclude, the prevalence of MCI and dementia found in this study was higher than that reported for Latin America. A low education level in our older population appears to be the main risk factor for cognitive impairment and should be seriously considered by our health care system. These recent studies from Latin America suggest that the epidemic of dementia is more serious than previously thought, and that the economic calculations for its prevention and management should be reviewed in our health systems.
